# Enhancing critical raw materials recovery in micro e-mobility: Regulatory strategies for the EU

**DOI:** 10.1177/0734242X251369685

**Published:** 2025-09-11

**Authors:** Topi Turunen, Claire Mosoni, Petrus Kautto

**Affiliations:** Finnish Environment Institute, Helsinki, Finland

**Keywords:** Circular economy, micro e-mobility, recycling, critical raw materials, electronic waste, waste

## Abstract

Novel electrical mobility solutions such as micro e-mobility devices (e-bikes and e-scooters) are rapidly increasing in modern societies. Currently, research on the theme has not focused on the circular economy of the devices and critical raw materials (CRMs) incorporated in their components has not received a lot of attention. In particular, existing regulations regarding the end-of-life management of the devices have, to date, been insufficiently explored or studied. CRMs can be found in devices’ batteries, electronic motors and printed circuit board. Their recovery is crucial from the point of view of European Union’s (EU’s) objectives regarding circular economy and self-sufficiency of raw materials. However, the current regulatory means implemented by the EU provide limited incentives for more efficient CRMs recovery in micro e-mobility. This article discusses possible policy innovations as means to address the shortcomings of the EU regulations. These include setting out recovery targets for CRMs and requirement for recycled CRMs content for electronic products through amendments of the waste electrical and electronic equipment directive and the ecodesign framework. Nonetheless, the EU faces a challenge in increasing the capacity for the recovery of CRMs and CRMs’ rich waste streams.

## Introduction

Electric mobility represents a global trend that is gaining foothold in modern societies, alongside urbanization, digitalization and green energy. A specific subset of this trend, micro e-mobility, has experienced a rapid expansion, with for example, shared e-bikes now present in over 600 cities across more than 50 countries worldwide (Liu et al., 2023). Micro e-mobility corresponds to two- and three-wheeled small mobility devices powered by electricity, such as e-bikes and e-scooters.

Currently, research on the micro e-mobility sector has mostly focused on five main themes: the energy system of e-micro-mobility, traffic behaviour and safety rules, carbon emission, health benefits and e-micro-mobility usage compared to other transport modes (Guy et al., 2021; Homem de Gouveia et al., 2023; Zhou et al., 2023). Despite an increasing fleet worldwide, little attention has been given to end-of-life of micro-e-mobility (Baumgartner and Helmers, 2024).

At the same time, the amount of waste electrical and electronic equipment (WEEE) generated each year has been increasing in Europe (European Commission, 2016). The growing amount of micro e-mobility poses an additional risk of accelerating the growth of European WEEE streams. The European Union (EU) has enacted ambitious circular economy objectives (COM, 2020b) including measures to harness waste materials as substitutes for virgin raw materials. In recent years, the EU has become increasingly aware that certain materials are more crucial to recover than others, known as critical raw materials (CRMs). These materials are essential for the EU’s industry, yet the EU is heavily reliant on imports from a limited number of suppliers in third countries, which could present a significant risk in the future (COM, 2020a, annex 1; European Commission, 2023). From this perspective, questions about the recovery of CRMs from decommissioned micro e-mobility devices are highly relevant.

This article looks at the existing policies and legislation regarding the recycling and circularity of CRMs from micro e-mobility devices. We answer to following questions:

- How is the end-of-life of the micro e-mobility devices regulated in the European Union? Are there tangible obligations to promote the recovery of CRMs from its different electric components at the end of its life?
- What kind of policy innovations can be adopted in order to enhance more sustainable CRM management from micro e-mobility devices?

The scope of the article is limited to a single product category, which is considered increasingly important and understudied, but most of the findings are applicable for many other electronic products. Indeed, many different CRMs (e.g. antimony, beryllium, cobalt, germanium, indium, natural graphite, rare earth elements, silicon metal and tungsten) are extensively used in electrical and electronic equipment (EEE) and could be recovered from their waste (Mathieux et al., 2017).

In this article, policy innovations which could be adopted to recover CRMs from micro e-mobility refer to new ways to address to an old or a newly found problem (Kautto and Valve, 2019; Schoenefeld et al., 2022; Weir, 2019). Innovativeness of the policy is naturally time and place dependent: A policy that has been used in one context can be innovative when applied to another (Fromond et al., 2009; Patterson, 2021).

The article unfolds as follows: firstly, it briefly describes the methodology of the study. Secondly, it presents the electronic components of the devices and the valuable materials which can be recovered from them. Thirdly, the article examines the existing EU legislation affecting their recovery, especially focusing on the legal provisions governing the end-of-life of the different components of the devices. Fourthly, the article identifies and discusses relevant policies and policy innovations that could be utilized to increase the recovery rate of CRMs from the devices.

## Materials and methods

**Figure 1.**
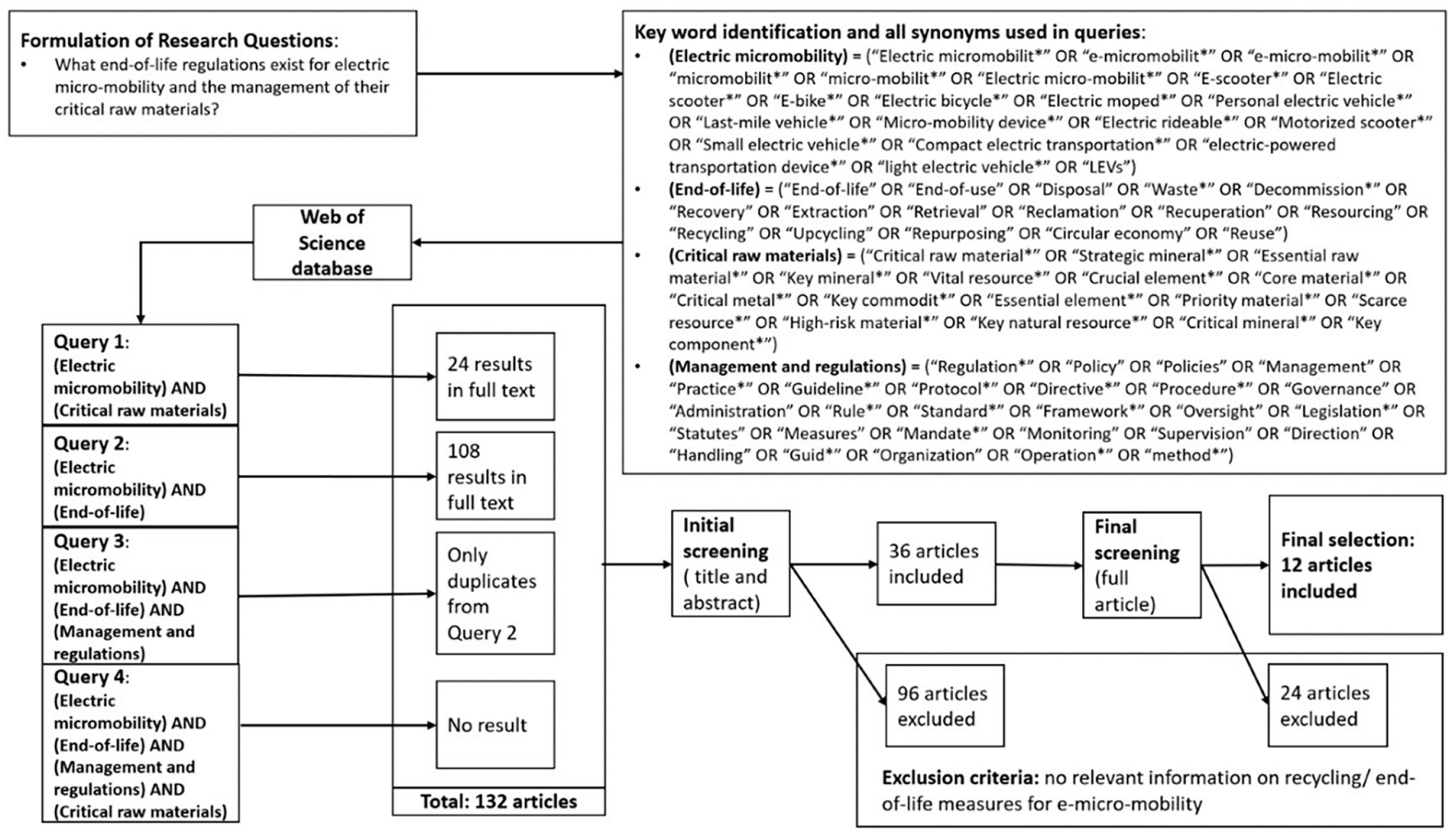
Literature review process.

As a first step, the scientific literature on end-of-life electric micro-mobility and CRMs management was scrutinized by doing a systematic literature review, using the same method as Merli et al. (2018) and Shaffril et al. (2021). The result of the systematic literature review show, most of all, the lack of literature on this particular topic. The search in full articles that would encompass terms related to ‘regulations’, ‘end-of-life’, ‘e-micro-mobility’ and ‘CRMs’ yield no result and the search had to be broaden to only account for some of the terms used in our systematic literature review (Figure 1). The consequence of this was a final selection of articles related to our particular subject to only account for 12 articles out of the original 132 articles from the search. Most articles focused on the future recycling demands, the recycling potential and innovative recycling methods of batteries, permanent magnets or generally micro e-mobility devices with limited insights on policies and regulations.

To gather more scientific literature following the low number of articles selected from the systematic literature review, a scoping review of the scientific literature on the recycling of different electric parts of micro e-mobility (batteries, permanent magnets and printed circuit boards (PCBs)) was also carried out.

Finally, a thorough analysis of the legislation regarding micro e-mobility devices and their recycling at the EU level was realized. As we only identified few legal instruments directly targeting CRMs and micro e-mobility, we also focus on instruments that impact the waste management of electronic fractions in general (under which micro e-mobility falls) as there is often an indirect impact towards CRMs recovery. The analysis was carried out through legal analysis and especially aimed at systemizing the regulatory framework consisting of the fragmented provisions. On the basis of the legal analysis and the literature review, we drafted proposals for policy innovations to better address the recovery of CRMs from end-of-life micro e-mobility devices in the future.

There are some limitations concerning the database used for the literature review: Searches that were conducted in Web of Science database mostly provide research articles and similar materials but do not necessarily describe the technological maturity of the recycling industry as closely as information gathered from the industries, patents and conference presentations. Nevertheless, as this article focuses on regulatory strategies for enhanced CRMs recovery, the literature review conducted offers a sufficient ground to build on.

## Micro e-mobility and CRMs

CRMs have been gaining more attention in recent policy discussions due to the estimated growth in their demand as well as increasing geopolitical tensions. The International Energy Agency (2021) predicts that, to meet the objectives under the Paris Agreement, the demand for copper and rare earth elements will raise 40%, the demand for nickel and cobalt 60–70% and the demand for lithium almost 90%. Due to this increase in demand, it has been assessed that known reserves of lithium, cobalt and nickel may be depleted by 2050 (Remme and Jackson, 2023).

In the recently adopted CRM Regulation (EU) 2024/1252, EU has classified 34 materials as CRMs. The Regulation introduces a subcategory of strategic raw materials (SRMs) to CRMs. SRMs are used to promote the green transition, digitalization and defence industry technologies. Their availability suffers from a gap between global supply and demand (including, e.g. cobalt, copper, lithium). In addition to SRMs, CRMs include other raw materials which are of economic importance to Europe and for which the risk of a supply disruption is high (e.g. hafnium, heavy and light rare earth elements, niobium).

EU is not self-sufficient in CRMs and is dependent on imports from third countries. It has highlighted material substitution and recovery to improve self-sufficiency and reduce impending supply risks (SWD, 2018). The recovery approach would establish a secondary reserve of recovered CRMs, diminishing dependency on imports. Nevertheless, it remains unclear whether measures geared towards that end have been implemented in the relevant legislative framework (Turunen and Suikkanen, 2024), as the recovery rate for most CRMs remains low (0–17%). Some CRMs have higher recovery input rates (28–44%), examples being vanadium, tungsten, cobalt and antimony (European Commission, 2018). The EU CRM Regulation sets an ambitious benchmark of producing at least 25% of the Union’s annual consumption of SRMs through recycling by 2030.

**Figure 2.**
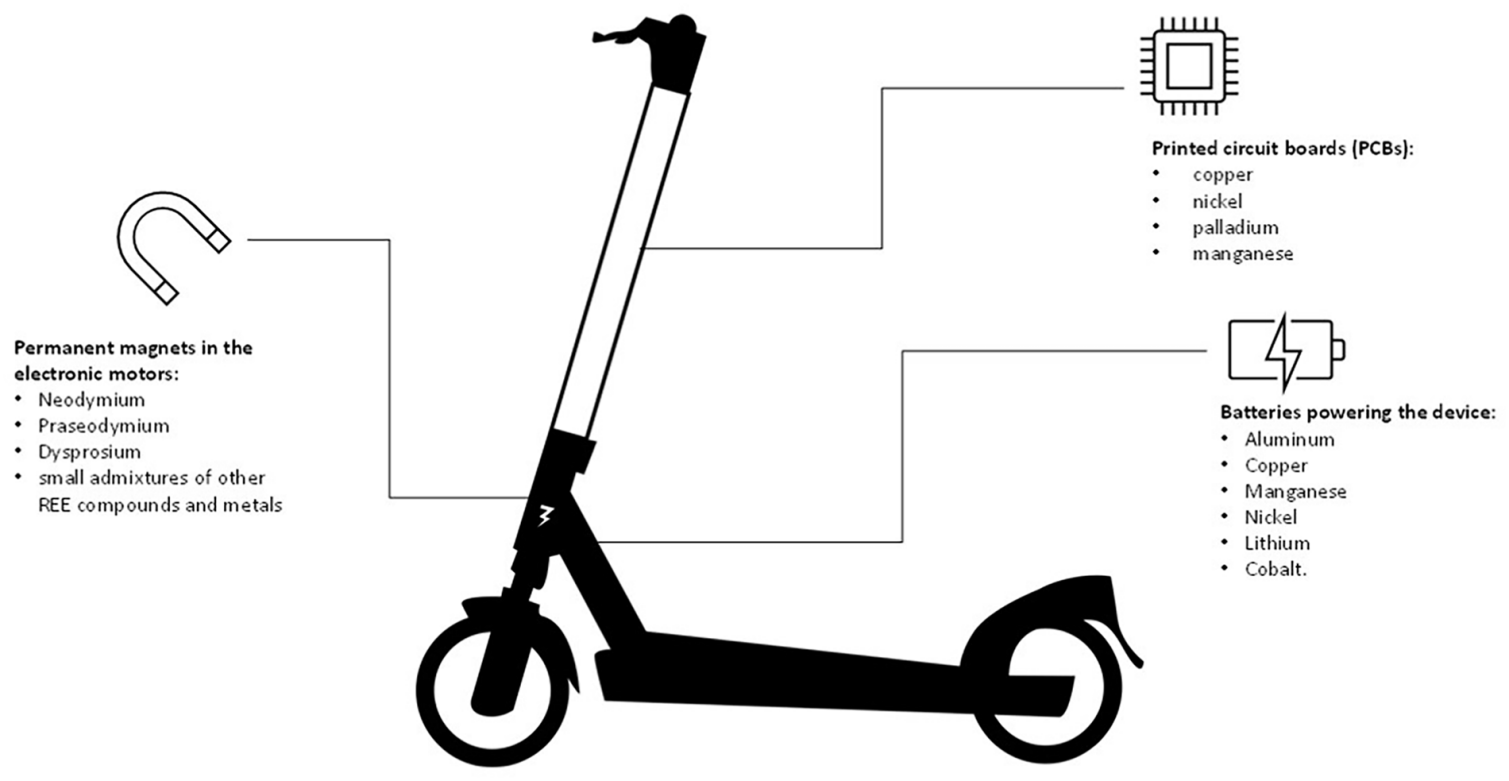
Critical raw materials incorporated in the components of an e-scooter.

CRMs are key materials of micro e-mobility devices, located in the batteries, permanent magnets and PCBs (Figure 1). The lithium-ion batteries of e-bikes and e-scooters include aluminium as a CRM as well as the following SRMs: copper, manganese, nickel, lithium and cobalt (Bobba et al., 2020; Turkki et al., forthcoming). In addition, neodymium–iron–boron permanent magnets commonly used in the devices include different heavy and light rare earth elements such as neodymium, praseodymium, dysprosium and boron (Schultz et al., 1999; Turkki et al., forthcoming). Lastly, different CRMs can be found in PCBs, which is a common component of almost all electronic device with variation in their CRMs content depending on their use. For micro e-mobility devices, the following SRMs and CRMs have been identified in their PCBs: copper, nickel, palladium and manganese (Figure 2; Cui and Anderson, 2020; Tuncuk et al., 2012; Turkki et al., forthcoming).

The rapid development and the resulting accumulation of CRMs waste from micro e-mobility devices in the urban environment make these devices a product with a high potential for CRMs recovery (Zhao et al., 2024).

Following is a simplified description of the most important recycling technologies that are currently available. This manuscript does not aim to determine the winning recovery technology or combination of technologies but focuses examination of the potential regulatory pathways towards enhanced recovery of CRMs. Ultimately, the role of the legislator is to pick the best policies to promote CRMs recovery while maintaining technological neutrality. Furthermore, it should be taken into account that many costly steps such as waste collection, stockpiling and sorting are necessary in order to get to the recycling process.

Currently, CRMs are mainly recovered through pyrometallurgy or hydrometallurgy processes. Pyrometallurgy consists of a high temperature treatment of the waste, separating the metals. It is an efficient method to recover valuable metal resources such as cobalt or nickel via metal alloys (Pražanová et al., 2024). However, pyrometallurgical treatments are energy intensive, costly and generate hazardous gases (Tuncuk et al., 2012). In addition, lithium is typically not recovered with this technique (Pražanová et al., 2024). Hydrometallurgy is based on acid leaching followed by the precipitations of the valuable metals (Bruno and Fiore, 2023). Hydrometallurgical processes offer high metal-rich recoveries, lower environmental impacts in terms of emissions (at the expense of consuming significant chemical reagents) but are mostly suitable for relatively small-scale applications (Behnamfard et al., 2013; Syed, 2006).

The recovery of CRMs in lithium-ion batteries is typically carried out through dismantling and dissolving the shredded material. This pre-treatment phase enables elimination of impurities and unwanted materials and reaching the highest-quality material called ‘black mass’ before separating and recovering the metals through the pyrometallurgy or hydrometallurgy techniques described above (Meng et al., 2021; Turkki et al., forthcoming). Due to the number of materials and complex structure of the batteries, pre-treatment can be quite difficult, with a lack of well-defined design (Pražanová et al., 2024). Furthermore, current recycling technologies to recover lithium are insufficient to manage the predicted amount of lithium waste in the future (Guo et al., 2021).

For permanent magnets, there are three main options: direct reuse, raw material recovery (pyrometallurgical or hydrometallurgical treatments) and alloy reprocessing. The direct reuse is often not possible due to developing technology and the fact that the dismantling of magnets is difficult. The recycling route consists of collection, demagnetization, fragmentation, classification and magnetic separation (Becci et al., 2021; Burkhardt et al., 2023; Cherkezova-Zheleva et al., 2024). However, the magnets only present a small part of the electronic product and the current efforts needed to recover CRMs from magnets may not be cost-effective (Lixandru et al., 2017). In particular, the collection phase needs to be improved to fully allow CRM recovery from permanent magnets (Burkhardt et al., 2023). Finally, reprocessing of the alloy requires magnetic waste with a known and homogenous composition and the thorough removal of possible impurities, which can make it hard (Elwert et al., 2017; Turkki et al., forthcoming). The pre-treatment phase needed to recover CRMs from PCBs is even more intricate as PCBs have complex and variable material composition. Nevertheless, the precious and critical materials in waste PCBs can make them an attractive waste stream for recovery.

## Policies promoting the recovery of CRMs

### Introduction

This section delves into the current policies on the recovery of micro e-mobility devices. The current regulatory framework consists of various provisions with diverse effects on the recovery of CRMs from the end-of-life devices.

Different legal lenses can be taken towards micro e-mobility devices. Typical micro e-mobility devices do not qualify as vehicles under the EU law. Therefore, the Directive on End-of-Life Vehicles (2000/53/EC) is not applied to them. Instead, the devices correspond to EEE, their waste falling under the scope of application of the WEEE Directive (2012/19/EU; European Commission, 2013).

As the CRMs incorporated in the micro e-mobility devices are mostly used in batteries, permanent magnets and PCBs, policies on CRMs recovery in each of these components are scrutinized. We assess whether these policies need to be improved or complemented with new instruments for promoting circular economy of the micro e-mobility devices.

### Recovering CRMs from the batteries

The batteries powering the micro e-mobility devices (mainly lithium-ion) are one of the key components. As stated earlier, batteries contain many CRMs and their demand is predicted to skyrocket in the coming years (Gianvincenzi et al., 2024). The Batteries Regulation (EU) 2023/1542, enacted in 2024, replaces the EU Batteries Directive and has multiple provisions concerning the recovery of CRMs from batteries. Regulation aims to rectify the past shortcomings by addressing, for example, the increasing demand for lithium-based batteries and their environmental challenges (Penttinen, 2021).

In the Batteries Regulation, a new category of light means of transportation (LMT) batteries is introduced. LMT batteries are defined as batteries that are sealed, weighs 25 kg or less and is specifically designed to provide electric power for the traction of wheeled vehicles that can be powered by an electric motor alone or by a combination of motor and human power and that is not an electric vehicle battery. Regulating LMT batteries separately was seen necessary due to their increased sales and the fact that they have a longer lifetime than portable batteries (Recital 108 of the Regulation).

Producers of LMT batteries are required to reach the following collection targets (a) 51% by end of 2028 and (b) 61% by end of 2031. Member States must enforce these targets and monitor collection rates to verify that adequate measures have been taken. The Regulation also lays down the following recycling targets for batteries: by the end of 2025 – (a) 75% of lead-acid batteries (which used to be widely used in micro e-mobility), (b) 65% of lithium-based batteries (currently the most used for micro e-mobility), (c) 80% of nickel-cadmium batteries and (d) 50% of other waste batteries. By the end of 2030, the recycling target for lithium-based batteries rises to 70% and the recycling target for lead-acid batteries to 80%. These recycling targets are weight-based, meaning they do not separate between the different materials that are recovered. The Batteries Directive had similar general recycling targets.

The Regulation lays down additional recycling target directed specifically to CRMs in waste batteries. The following material-specific recycling targets for all waste batteries are given: (a) 90% for cobalt by the end of 2027, 95% by the end of 2031, (b) 90% for copper by the end of 2027, 95% by the end of 2031, (c) 90% for lead by the end of 2027, 95% by the end of 2031, (d) 50% for lithium by the end of 2027, 80% by the end of 2031 and (e) 90% for nickel by the end of 2027, 95% by the end of 2031.

In addition, the Batteries Regulation states that from 18 August 2036, LMT batteries that contain cobalt, lead, lithium or nickel in active materials shall contain information about the percentage of these materials that have been recovered from waste. It also stipulates the following recycled materials content requirements: (a) 26% cobalt, (b) 85% lead, (c) 12% lithium and (d) 15% nickel. These targets may be revised later based on the existing or forecasted availability of cobalt, lead, lithium or nickel recovered from waste, taking into account the technical and scientific progress. Furthermore, European Commission can add new materials with specific minimum shares of recycled content where justified and appropriate due to market developments regarding battery chemistries.

One of the key elements to reach these objectives is implementing mandatory Extended Producer Responsibility (EPR) schemes for batteries. Producers of LMT batteries ensure that all waste LMT batteries are collected separately in the territory of a Member State where they make batteries available on the market. To organize this, they have to establish a take back and collection system, offer free collection and arrange their collection and transport for waste batteries collected at the connected collection points and removed from WEEE streams. After collection producers need to ensure that batteries are subject to adequate treatment (including reaching the recycling targets) in a permitted facility. Producers can appoint a producer responsibility organization (PRO) and transfer their obligation to the PRO. If the batteries are incorporated into an electronic product, the waste products that contain batteries will be handled as WEEE and the batteries will be separated in their selective treatment (see section ‘Recovering CRMs from the printed circuit boards’). After separation, the distributors shall hand over waste batteries to the producers or PROs or to a waste management operator.

One significant addition in the Batteries Regulation is the battery passport. The battery passport is an electronic record that is introduced to increase the transparency of the battery value chain (Penttinen, 2021) and to encourage informed business decisions and help battery recycling. LMT batteries shall be marked with a QR code that provides access to the battery passport. From 18 February 2027, each LMT battery placed on the market or put into service shall have a battery passport. The passport would contain information relating to the battery model and information specific to the individual battery including, for example, information on the composition and CRM content of the battery and the regular performance. This would facilitate the recovery of the CRMs from the batteries by helping to identify valuable materials and substances of concern and their potential as resources in terms of quantities and quality (Alaranta and Turunen, 2021; COM, 2018; de Römph, 2018). The Batteries Regulation also regulates the removability and replaceability of LMT batteries by implementing the obligation to remove or replace them by a professional at any time during the lifetime of the product.

### Recovering CRMs from the permanent magnets

The WEEE Directive does not provide requirements concerning electronic motors or permanent magnets. Certain provisions concerning CRMs and permanent magnets are laid down in the EU CRM Regulation. However, its main emphasis is not on the circular economy of CRMs but more on ensuring the supply of virgin CRMs in the EU (Turunen and Suikkanen, 2024).

According to the CRM Regulation, when a light means of transport device containing a permanent magnet is placed on the market, it has to bear a label indicating that it incorporates permanent magnets and stating whether the magnets belong to any of the following types: neodymium–iron–boron, samarium–cobalt, aluminium–nickel–cobalt or ferrite. Moreover, there needs to be a data carrier on or in the product carrying the information on, among other things, the permanent magnets included in the product. Furthermore, in case of products incorporating permanent magnets with a total weight of more than 0.2 kg, the share of recovered neodymium, dysprosium, praseodymium, terbium, boron, samarium, nickel and cobalt present in the product should be made public. According to the study by Turkki et al. (forthcoming), neodymium–iron–boron magnets were used in all the studied micro e-mobility devices, and the masses of the magnets were ranging from a little under and well over the threshold of 0.2 kg. Hence, most of the requirements above should be applied to permanent magnet in the devices in micro e-mobility devices. However, the provisions concerning permanent magnets do not issue very strong obligations currently. The provisions of the CRM Regulation mainly aim to increase the amount of available information regarding the presence of permanent magnets and CRMs in them.

Most of these CRMs are SRMs listed under ‘Rare earth elements for magnets’ in the Regulation. For these particular rare earths, the Commission shall adopt, by the end of 2031, delegated acts laying down requirements stipulating the minimum amounts of recycled material that must be used in the manufacture of permanent magnets incorporated in the products, thus strengthening the current provisions. The minimum share of recycled CRMs in the magnets would be based on a prior impact assessment on the functioning of products incorporating permanent magnets and taking into account the availability of recovered CRMs.

Moreover, Regulation (EU) 2019/1781 could be relevant for the recovery of CRMs from permanent magnets. It lays down performance standards for electric motors and as part of its regular revision, it should address the appropriateness of setting additional resource efficiency requirements for products, including identification and reuse of rare earth in permanent magnet motors. However, it is unclear what kind of instruments would be included in the revision of this Regulation.

### Recovering CRMs from the PCBs

From the three main electronic components of micro e-mobility devices, PCBs have been addressed the least in legal interventions. There is no separate regulatory framework for PCBs. The most relevant provisions affecting their recovery come from the general provisions concerning EEE and WEEE.

Indeed, PCBs would fall under the weight-based recovery targets for collected WEEE, not taking into account the different materials within the waste streams (Horta Arduin et al., 2020; Ortego, 2018). Depending on the product category, the recovery targets range from 55% to 85%. In annex I of the WEEE Directive two-wheel e-mobility devices that are not type-approved belong to category 7 (toys, leisure and sports equipment). That means the recovery target for them is 75%. To reach these recovery targets, the Directive sets out an EPR scheme for WEEE. However, the targets focus on calculating the bulk volume of recovered waste and might not lead to an optimal outcome for CRMs recovery, as CRMs are usually utilized in small quantities and the products often contain smaller quantities of CRMs than of other materials (Hagelüken, 2014; Penttilä, 2020; van Ewijk and Stegemann, 2016).

In addition to the EPR and weight-based recovery targets, the Directive lays down minimum rules on proper treatment of WEEE. These include selective treatment for materials and components listed in Annex VII of the directive. PCBs are one of the components listed, with a requirement to separate the PCBs from the rest of the device if the surface of the PCBs is greater than 10 cm^2^. Notably, permanent magnets are not listed to be removed in the selective treatment. The PCBs in electronical devices differ significantly in size and the requirement for selective treatment is not applied to all PCBs of micro e-mobility devices. For the bigger PCBs, the selective treatment requirements provide more homogenic material fraction for the recovery operators. The WEEE Directive is currently under review and amendments are expected to take place in the near future.

## Discussion: Policy innovations for better recovery of CRMs

The growing fleets of micro e-mobility devices raise questions regarding the materials that are used in them and the circular economy of those materials. Much like many other electronic products, the e-mobility devices are complex products that contain many different CRMs in their components. The recovery of these CRMs is important to break from the ‘take-make-dispose’ economy approach and unsustainable use of non-renewable resources. As the EU is currently dependent on global importations of CRMs, stricter governance on the end-of-life recycling of these materials is a logical step to reduce the risks related to the global competition for resources. The unhindered access to CRMs is vital for the green and digital transition of the EU (COM, 2020a).

In this article, we examined the current policy instruments promoting the recovery of CRMs from different components of a micro e-mobility device. Suffice to say, the current policy framework is not equipped to promote efficient recovery of CRMs from these devices. The analysis of the applicable legislative instruments on the EU level shows that beyond the new Batteries Regulation, very little emphasis has been put on promoting the recovery of CRMs in other components of micro e-mobility devices. The same applies to most electronic products.

In any case, the management of CRMs in EU legislation is a relatively new trend despite long-standing recognition of the problems associated with their use and availability (Turunen and Suikkanen, 2024). The linear economy is embedded in the current regulatory framework and as the obligations and incentives for the recovery of CRMs are mostly missing and additional tangible obligations for Member States and private actors are necessary. For example, CRMs are in most cases used in small quantities in EEE compared to overall weight of the product and its components and weight-based recycling target laid down in the WEEE Directive do not effectively promote their recovery (Hagelüken, 2014; Penttilä, 2020; van Ewijk and Stegemann, 2016).

The new Batteries Regulation has taken steps forward with enacting more advanced recycling targets that focus on the recycling of the most important CRMs in batteries in addition to the weight-based ones. They offer more tangible obligations to promote the recovery of CRMs from batteries. Nevertheless, as recycling technologies evolve (Abdelbaky et al., 2021; Gianvincenzi et al., 2024) and transition towards fossil-free energy production moves on, also the WEEE Directive should stay up to date in its CRM recovery requirement with these fast-paced changes.

The Batteries Regulation lays down recycled content targets concerning CRMs in new batteries. Similar targets may be laid down by the Commission for permanent magnets under the CRM Regulation. The rationale of this kind of instruments is to create demand for recycled materials, and as such, promote recycling. For example, the targets for recycled CRM content in new batteries are deeply connected with the ambitious recycling targets for CRMs from decommissioned batteries. As the composition of EEE varies significantly, it may not be viable to lay down general recycled content requirements for CRMs under the WEEE Directive. Instead focus on could be put on specific electronic components, or specific products with high CRM content which are becoming increasingly important with the transition to a low-carbon economy, such as micro e-mobility devices.

This approach may be taken in the new ecodesign framework. The new ecodesign Regulation (EU) 2024/1781 has been accepted in the summer of 2024. The new provisions enable the regulation of a wide array of products, including micro e-mobility devices (which were specifically mentioned in Recital 13). This framework offers new ways to address the circular economy of micro e-mobility devices by providing opportunity to regulate the whole product at once instead of addressing its recycling through sporadic regulatory instruments which either focus on separate components or on a very wide product group such as EEE in general.

One of the key factors for the recovery of CRMs is identifying them in the products. Both the Batteries Regulation and the Ecodesign Regulation introduce a product passport scheme. As the product passport is a novel requirement, its full potential in promoting circular economy has not yet been realized, but it promisingly enables identification, tracking and acknowledgement of many different properties of the products. Additionally, product passports could help to raise public awareness of battery recycling (Chen et al., 2017).

Beyond the limitations of the current regulatory framework on CRM recovery, the technical and economic viability of the recovery operations has also raised questions. There are already functioning EPR schemes with collection and recycling routes for WEEE streams. These routes should be extended to include new capacities for CRMs recovery that would enable the recovery of CRMs from complex electronic products such as micro e-mobility devices. However, the lack of suitable processing and recycling facilities creates challenges for future recycling and recovery needs. For example, Bruno and Fiore (2023) argued that to reach better recovery rates of lithium recovery, EU needs to invest in more hydrometallurgy facilities instead of pyrometallurgy facilities which are unable to recover lithium. Moreover, the centralization of the recycling facilities can make transport costs of the recovery system higher and hinder its economic viability (Rallo et al., 2022). According to Gianvincenzi et al. (2024), CRMs in batteries are often lost as the black mass that is produced in the batteries recycling process and later exported outside the EU.

To increase the production, processing and recycling capacities of CRMs in EU, the CRM Regulation introduces a streamlined permitting process for strategic projects. Strategic projects enjoy a faster and easier permitting procedures and are prioritized as projects of public interest in certain exemptions of substantive environmental law. The operators may apply for a strategic project status for their operation under different conditions (meaningful contribution to the security of supply of SRMs, technically feasible, sustainable, beneficial beyond national borders, inclusion of downstream sectors and if third countries are involved, the project is mutually beneficial for both EU and the third country concerned). As this streamlined permitting process is a novel approach, its positive (increasing processing capacity in EU) and negative (increased local impacts on the environment) outcomes remain to be seen (see Ala-Lahti and Turunen, 2025).

New requirements for enhanced CRMs recovery already put the pressure on growing the capacities for CRMs in the EU. If further requirements were to be introduced, the demand for new capacity would be even bigger. Although introducing more ambitious requirements for CRMs recovery is necessary, adequate transition periods should be implemented in order to ensure sufficient processing capacity to reach the targets. In addition to the streamlining efforts of the CRM Regulation, the uptake of new recycling capacity can be supported with different kinds of public–private funding and investments.

For example, sorting methods for retired lithium-ion batteries need to be improved in recycling facilities, as it would allow to repurpose and remanufacture more batteries, which may retain over 60% of their original capacity and are able to be used in the small electric vehicles that corresponds to micro e-mobility (Phophongviwat et al., 2023). Some approaches on how to improve the sorting methods include X-ray computed tomography images (Ran et al., 2020).

## Conclusions

The demand for CRMs is expected to skyrocket in the near future. The EU’s aim for green transition together with the growing geopolitical instability creates an urgent need to improve CRMs management in Europe. At the same time, the fleets of micro e-mobility devices are growing rapidly. The recovery CRMs from end-of-life micro e-mobility devices or other complex electronic products has not been extensively studied before, especially from a policy standpoint. This study focused on the three components that were identified as being most relevant for the recovery of CRMs from micro e-mobility devices: their batteries, permanent magnets incorporated in their electronic motors and PCBs in the devices.

The article analyses the regulatory instrument that have been laid down in the EU and assesses what kind of policy innovations could fill in the gaps of the current regulatory framework and promote the recovery of CRMs from micro e-mobility devices. These include setting out CRMs’ specific recovery targets and recycled content requirements for electronic components in the revisions of the WEEE Directive and regulating the product design through the Ecodesign Regulation.

Nonetheless, even with the strengthening of policy instruments in place, the EU faces a challenge in increasing the capacity for the recovery of CRMs and CRMs’ rich waste streams. Streamlining permitting and promoting new investments in EU strive to address this issue but may not be sufficient, calling for new funding instruments to effectively promote the uptake of new recycling capacity.

Crucial questions and research gaps still remain. Firstly, there is a need for more research on what happens at end-of-life of micro e-mobility devices in practice, especially if their fleets keep growing. There are still too many uncertainties concerning which technologies, CRM compositions of the devices and dismantling processes will prevail in the future and how it will influence the recycling process and recycling facilities. This situation makes the efficiency of the current recycling system nowhere near where it should be to allow the breadth of CRM needed for the green transition. Therefore, more investments on research and development of technologies and recycling methods are urgently needed in the micro e-mobility sector.
